# Supplementary material for: Identification and initial characterization of Hfq-associated sRNAs in Histophilus somni strain 2336
Source: PLoS One. 2023 May 23;18(5):e0286158. doi: 10.1371/journal.pone.0286158 (PMC10204968; doi:10.1371/journal.pone.0286158)
Supplement: S1 File — (DOCX) [file pone.0286158.s001.docx]

**S1 Supporting Information**. Criteria for the identification of sRNAs

The mapping of the sRNA reads to *H. somni* strain 2336 reference genome was performed by Geneious prime. The aligned RNA-sequence based transcriptome map was manually screened to identify intergenic regions. The intergenic regions were also compared with those previously reported for *H. somni* strain 2336 [reference 8]. Promoters and terminators were predicted to confirm the identified transcripts (intergenic regions) using the online tools BPROM (Softberry Inc., Mt. Kisco, NY) and ARNold [reference 30], respectively. To increase the specificity, BPROM was run on the sequence upstream of the identified transcripts within the region between two ORFs. BPROM was run with the default settings. ARNold finds rho-independent terminators in nucleic acid sequences. The search procedure used two complementary programs, Erpin and RNAmotif. The intergenic region between two ORFs was considered for identifying the rho-independent terminator and the default settings for the prediction tool was applied.

(A)


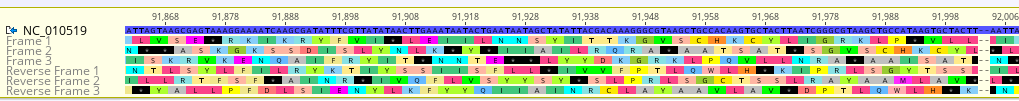


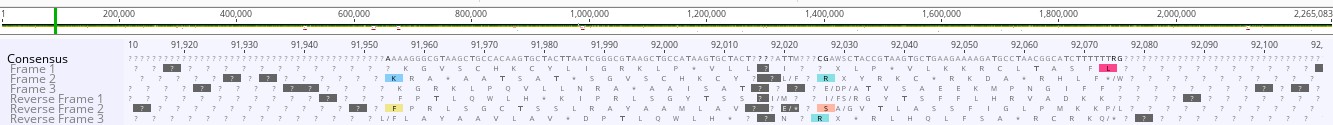
(B)

**S2 Supporting Information for Fig1.** (A) Representative zoomed image of the six-frame translation (forward three frames and reverse three frames) of the *H. somni* 2336 genome reference sequence. The six-frame translation of the reference sequence refers to the translation of the *H. somni* genome in three overlapping reading frames in the forward direction and the complementary strand in the reverse direction. (B) Representative zoomed image of the six-frame translation (forward three frames and reverse three frames) of the consensus sequence. The consensus sequence shows which residues are conserved (are always the same) and which residues are variable once we align the sequence to the reference genome sequence. The consensus is constructed from the most frequent residues at each site in the alignment.
