# Supplementary material for: Identification and initial characterization of Hfq-associated sRNAs in Histophilus somni strain 2336
Source: PLoS One. 2023 May 23;18(5):e0286158. doi: 10.1371/journal.pone.0286158 (PMC10204968; doi:10.1371/journal.pone.0286158)
Supplement: S1 Raw images — (PDF) [file pone.0286158.s002.pdf]

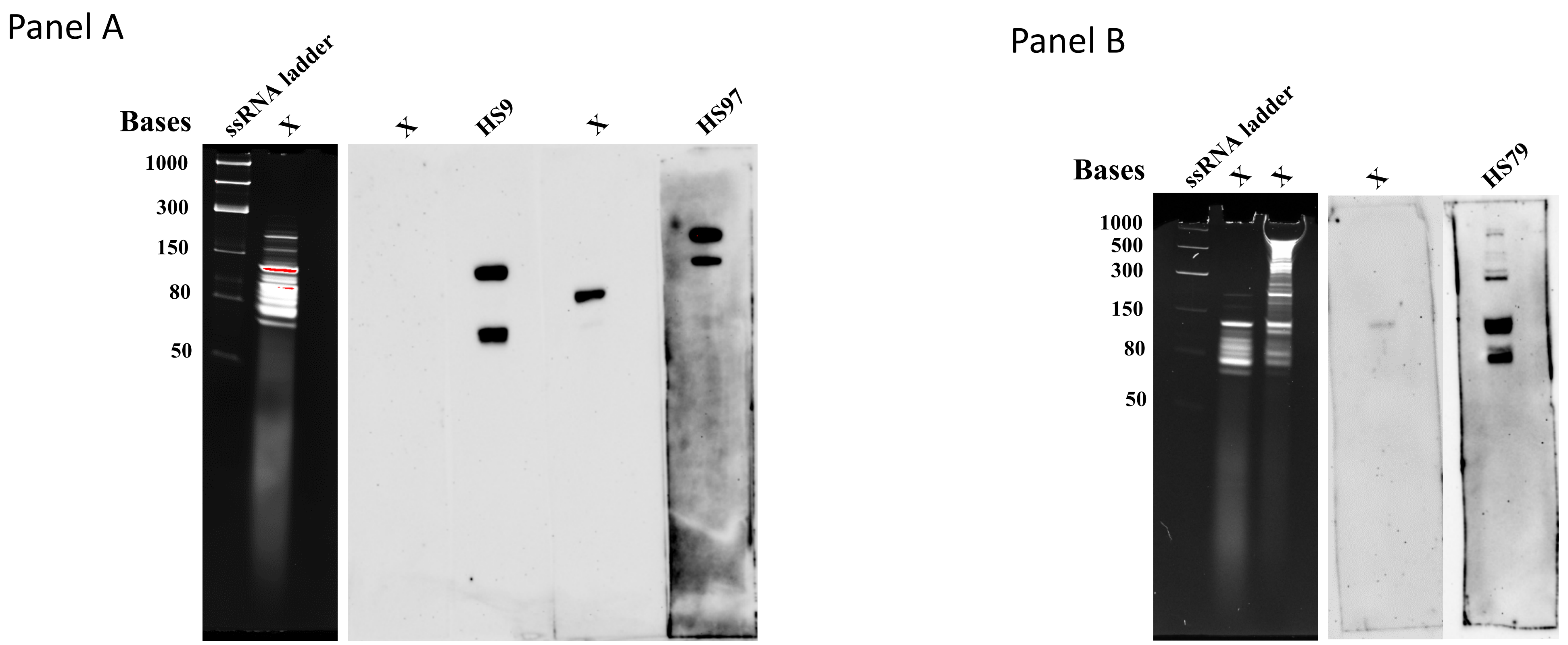

Fig 3. Northern blot. For Northern blot, the RNA samples along with the markers in each panel were loaded into the same gel. After gel electrophoresis, RNA samples were transferred to blotting membrane. Same conditions were used for pre-hybridization, hybridization, signal development and image capture for all the samples.

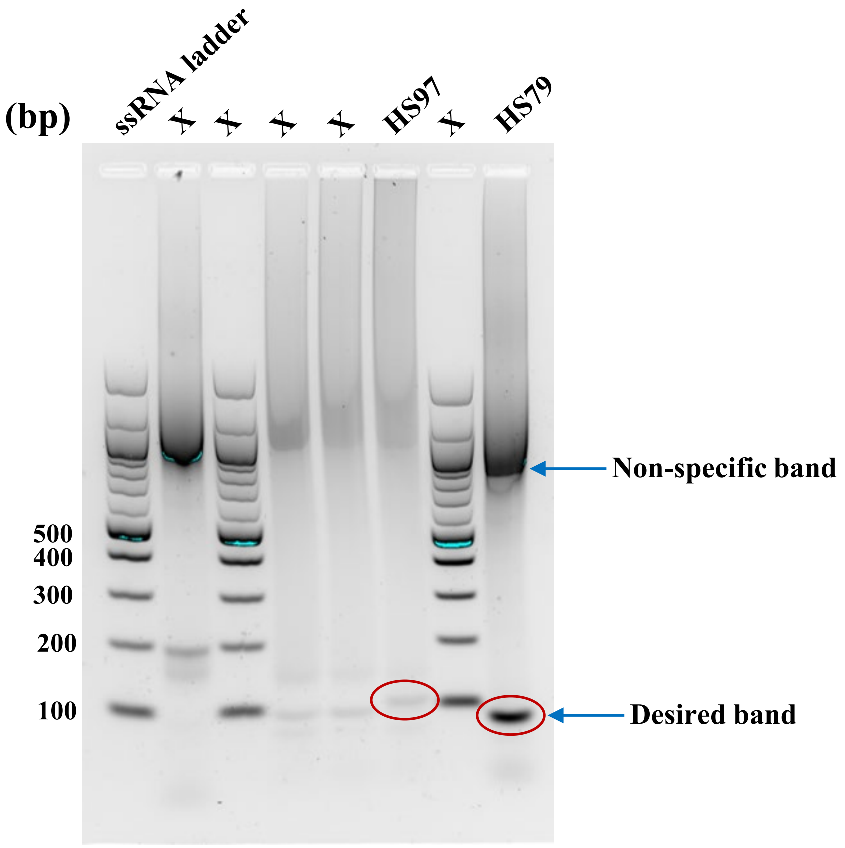

Fig 4. Identification of the transcriptional start sites of sRNAs HS79 and HS97 by RLM-RACE. (A) Identification of the transcriptional start sites of sRNAs HS79 and HS97 by RLM-RACE. The DNA bands considered for cloning and sequencing are circled. The whole Fig. 4A was created from this gel image.
