# Supplementary material for: Identification and initial characterization of Hfq-associated sRNAs in Histophilus somni strain 2336
Source: PLoS One. 2023 May 23;18(5):e0286158. doi: 10.1371/journal.pone.0286158 (PMC10204968; doi:10.1371/journal.pone.0286158)
Supplement: Attachment — Submitted filename: Responses to Reviewers.docx [file pone.0286158.s003.docx]

Reviewer #1: This is a well performed study that describes a set of sRNA molecules in *Histophilus somni* that were identified as interacting with the RNA chaperone Hfq.
While there are no major comments about the experimental design or data, this reviewer feels that the manuscript leaves the reviewer hungry, raising many unanswered questions. Solving this would need a considerable amount of additional experimental work. It is suggested that the authors make the manuscript more concise and direct by shortening the methods and that some of the speculative parts of the results and discussion are also reduced.

Thank you for each of your valuable suggestions. We have shortened the methods, results and discussion sections to make the manuscript more concise.

Minor comments, corrections and suggestions for shortening the text
1 Is Fig 1 really needed? Also, the title is not correct, this is ‘detection’, not ‘identification’.

We have removed this figure and changed the title to begin with “Detection”.

2 IntaRNA does not indicate or show that a sRNA can bind to a gene, it suggests or predicts this. Please change this in the text, or show experimental evidence.

We have taken out this paragraph.

3 There is an error in Fig5, why is the panel with uspE in the centre shown twice

We have corrected now Fig. 7 (new figure no.).

4 Are there any sequence motifs that are common to the multiple targets that could explain the promiscuous targeting of these genes numerous RNAs?

The majority of the target genes were binding to the typical A/U-rich regions found in small RNAs. It has been reported that the sRNAs can interact with multiple target genes, and their interaction is through the internal A/U rich regions and the 3’ U-rich region after the transcriptional terminator (Andrade JM et al. 2013, PlusOne). In addition, some sRNAs interacted with their target genes at the UG-rich regions and the role of UG-rich regions in binding to target mRNAs has been reported earlier (Lukavsky PJ et al. 2013, Nat. Struct. Mol. Biol.). I have added this information with the references in the revised manuscript.

5 For section 3.2. (Transcriptome-wide analyses of RNA sequencing results); ‘transcription wide’ is not appropriate, this is mapping of identified RNA sequences to the Hs genome.

We have corrected the statement in the text.

6 Fig 4 shows that some of the suggests that there are either alternative start sites for these molecules, or that they are processed. The authors write that RLM-RACE ‘only amplifies the primary transcripts, and not the processed products’. For HS97, why is the longer (so unprocessed?) not identified by this technique (L104, Fig 6). Do the authors have RNAseq data from this strain, which will allow them to determine whether the different transcripts are found? If not, they should mine the data from ref 8.

We referred to the paper suggested by the reviewer; however, we couldn’t find any information on the longer different transcript at the concerned location. However, an additional transcriptional start site upstream of the start site identified by RLM-RACE was predicted by the online tool, which is indicated by ‘arrow’ in Fig. 4C (new figure no.). We have added information about the additional transcriptional start site in the text, and also have modified the legend for Fig. 4 (new figure no.).

7 What do the RNA secondary structures bring to the manuscript? Here, experimental work is needed to show that this is of importance.

We included the predicted secondary structure of selected sRNAs as part of the preliminary analysis. However, the AU-rich regions in the secondary structure of sRNAs are also indicative of the binding sites for Hfq and other target genes, and therefore important.

8 The methods can be shortened; many of the procedures are standard or have been described in detail in previous work by the authors, including the overexpression and purification of Hfq, producing the antibodies, pulldown and purification of Hfq associated RNA, in vitro transcription and northern blotting (here I assume that you mean a 15% acrylamide gel and not a 15% Urea as indicated in the text!).

We have modified the methods session. We have deleted the detailed description of the standard protocols and given proper reference. Also, the previous paper by us detailing all the protocols was a pre-print version by Research Square.

Yes, 15% acrylamide gel. We have now specified this in the text.

9 The half page of conclusions is redundant.

We have made the ‘conclusion’ more concise.

Reviewer #2: In the manuscript “Identification and initial characterization of Hfq-associated sRNAs in *Histophilus somni* strain 2336” the authors outline a pull down procedure whereby they identify sRNAs that interact with the chaperone protein Hfq in the bacteria Histophilus somni. They go on to perform some initial characterization experiments on a select number of the identified sRNAs, including northern blots, mapping of transcription start sites, and confirmation of interactions with Hfq by EMSA.

Overall the work outlined is good, experiments are well controlled, performed appropriately and the manuscript is well written.

That being said, the study feels incomplete. The manuscript contains several sections that are based on computer predictions rather than experimental data and the amount of new experimentally generated data in the manuscript is relatively low. Follow up characterization is only performed for 2 or 3 sRNAs and no experiments are performed to investigate the role or function of any of them. The study would be enhanced if kore follow up work was performed on a larger number of the novel identified sRNAs.

Thank you for all of your valuable comments. We tried to characterize selected sRNAs to understand their role in various phenotypic and genotypic characteristics of *Histophilus somni*. We attempted to over-express the sRNAs using different vector systems suitable for this pathogen. However, over-expression of the sRNAs in *H. somni* was not successful, as this is a difficult pathogen to work with genetically due to a very tight restriction-modification system. In addition, the over-expression tools available for this organism are very limited. We are working on knocking out one or more of the more characterized sRNAs that are predicted to affect genes required for virulence. However, that will be a major manuscript in itself, and this work is an important step toward that goal.

In addition to the comments above the following issues need to be addressed.

Line 31: Please remove the sentence “to initiate understanding their role in regulation of virulence factors” as the role of these sRNAs in regulating virulence factors is speculative.

We have modified the text.

Line 36: replace the word “depicted” with suggests

We replaced the word ‘depicted’.

Line 64:… a class of regulatory RNAs ranging in size…” please insert “typically” in this sentence as sRNAs can be outside this size range

We have inserted ‘typically’ in the sentence.

Line 70 “…which plays crucial roles in a variety…”

We have modified it.

Line 101 The LB in LB medium stands for lysogeny broth not luria-bertani

We have corrected it.

Line 235: Please provide detailed information regarding the composition, size, and nature of the biotinylated probes used for northern blotting.

| **Sample** | **Barcode sequence** | **PF Clusters** | **% Perfect** | **% PF** | **% >= Q30** | **Mean Quality** | |
| --- | --- | --- | --- | --- | --- | --- | --- |
|  |  |  | **barcode** | **Clusters** | **bases** | **Score** | **Mapped Reads** |
| GSF2751-sRNA_Hfq-1 | CTATACAT | 4,416,420 | 100 | 100 | 88.01 | 32.85 | 633,747 |
| GSF2751-sRNA_Hfq-11 | TCGGCAAT | 2,667,639 | 100 | 100 | 88.69 | 33 | 18,160 |
| GSF2751-sRNA_Hfq-3 | CTCAGAAT | 5,000,014 | 100 | 100 | 88.25 | 32.9 | 427 |
| GSF2751-sRNA_Hfq-7 | GACGACAT | 3,411,220 | 100 | 100 | 88.82 | 33.02 | 1,633 |
| GSF2751-sRNA_Hfq-8 | TAATCGAT | 2,413,457 | 100 | 100 | 88.7 | 33 | 186,936 |
| GSF2751-sRNA_Hfq-9 | TCGAAGAT | 3,090,434 | 100 | 100 | 89.49 | 33.17 | 128 |

The sequence of the biotinylated probes were given in Table 1. We have modified the text under ‘Materials and Methods – Northern blotting’.

Line 270: Six independent sRNA preparations were used for deep sequencing yet on line 281 it states that two of the sRNA samples from which a large number of reads were obtained were considered for further analysis. Can the authors explain exactly how many samples were sequenced and how those datasets were processed? This is unclear.

A total of six sRNA preparations were used for deep sequencing. Out of the six sRNA samples, two samples (GSF2751-sRNA_Hfq-1 & GSF2751-sRNA_Hfq-8) having the highest number of mapped reads aligned to *H. somni* genome were considered for further analysis. The mapping of the reads to the *H. somni* genome was performed by Geneious Prime. We have added this information in the text.

The below table shows the RNA sequencing result summary.

Line 291: the authors should elaborate on the process that led to the identification of 100 sRNA candidates. What criteria were used, what cut offs were applied when analyzing the promoter and terminator predictions? This information could be included as a supplemental to the materials and methods.

The mapping of the sRNA reads to *H. somni* 2336 reference genome was performed by Geneious Prime. The aligned RNA-sequence based transcriptome map was manually screened to identify intergenic regions. The intergenic regions were also compared with those previously reported for *H. somni* 2336 [reference 8]. Promoters and terminators were predicted to confirm the identified transcripts (intergenic regions) using online tools BPROM (Softberry Inc., Mt. Kisco, NY) and ARNold [reference 30], respectively. To increase the specificity, BPROM was run on the sequence upstream of the identified transcripts within the region between two ORFs. BPROM was run with the default settings. ARNold finds rho-independent terminators in nucleic acid sequences. The search procedure used two complementary programs, Erpin and RNAmotif. The intergenic region between two ORFs was considered for identifying the rho-independent terminator and the default settings for the prediction tool was applied. The above information is included as a supplemental to the ‘materials and methods’ (labelled as S1 Supporting Information) as suggested.

Line 300: The figure legend for figure 2 needs to be greatly expanded. For example, please explain the significance of “six frame translation” and also the significance of the six black bars and one dark blue bar running from left to right across each figure.

The consensus sequence is displayed above the alignment or assembly, and shows which residues are conserved, and which residues are variable. The 6 frame translation of the consensus sequence is given just below it. The dark blue line indicates the reference sequence gene, and the six black lines below the blue line indicates the 6 frame translation of the reference sequence. The Fig 1 (new figure no.) legend has been elaborated.

Line 332: Please add more detail to the figure legend for figure 3. Indicate the significance of the color coding of residues. What is the significance of uppercase versus lowercase letters? The rho independent terminator T1 should be more clearly labeled. Specifically which residues are encompassed by T1.

All the positions in each sequence that are identical with the consensus are in upper-case, the other positions are in lower case. The residue that is highly conserved appears in high-consensus color which is red. A residue that is weakly conserved appears in low consensus color and is shown in blue. Other residues appear in neutral color which is black. A position with no conserved residue is represented by a dot in the consensus line. We have modified the figure legend and the T1 has been clearly marked in Fig. 2 (new figure no.).

Line 345: “A total of 8 sRNA candidates (HS9, HS14, HS26, HS72, HS79, HS86, HS97, HS98) were considered for further characterization based on preliminary bioinformatic analyses”. A detailed account of the selection criteria used to identify these 8 candidate sRNAs should be included. Why were these 8 selected. This information could be included as supplemental materials.

We selected a number of sRNAs which showed relatively high expression level in the sequencing data for further studies. Also, our initial plan was to do a comparative study by selecting a number of sRNAs conserved in phylogenetically closer bacterial genomes especially members of Pasteurellaceae family (*Mannheimia haemolytica*, *P. multocida*, Haemophilus *influenzae,* etc.) and also in distantly related bacterial species. Thus, the 8 sRNAs selected for further studies were belonging to those conserved in phylogenetically closer genomes or distantly related genomes. We have updated this information in the text.

Line 356: Please add the Sigma 54 consensus binding motif sequence to Table 3.

We have added the sigma 54 consensus binding motif 5'-TGGCACG-N4-TTGCW-3' to the Table 3 suffix.

Line 360: ” Based on the interaction with known genes that affect virulence and biofilm formation in Gram-negative bacteria…” What interactions and what genes are the authors referring to? The reasons for selecting these three sRNAs for further study are not clear. Furthermore, while 3 sRNAs were chosen for further analysis (HS9, HS79, and HS97) in some experiments data is only provided for two of them (HS79, and HS97 in Fig 6 and Fig 8) while in others there is data for all three (Fig 4, and Fig 7). In addition to explaining why these three were selected for further analysis please explain this inconsistency and/or provide data for HS9 in Fig 6 and Fig 8.

We were interested in identifying some sRNAs that are important for virulence and biofilm formation in *H. somni* for further studies. We checked the interaction of 8 sRNA candidates selected initially with the known biofilm/virulence related genes such as *luxR* (found two LuxR candidates in *H. somni*), *narQ*, *uspE* and *uspA*. Our group has reported that UspE is a global regulator controlling virulence and biofilm formation in *H. somni.* Finally, three of those sRNA candidates that interact with the above-mentioned biofilm/virulence genes were selected for further studies. Our initial plan was to overexpress two of the sRNAs in *H. somni* and characterize them by conducting phenotypic and genotypic studies. However, this approach did not work. Therefore, we concentrated on two sRNA candidates for the later part of experiments. We have modified the text.

Line 369: “…in the Northern blots indicated multiple transcripts…” replace “indicated” with “suggests”

We have changed it.

Line 363, Fig 4, and throughout the manuscript: When referring to the size of ssRNA molecules it is more accurate to refer to size in “bases” or “nucleotides (nt)” rather than “base pairs (bp)”.

We have corrected it as ‘bases’ throughout the manuscript and in figures.

Line 378: Target predictions using IntaRNA (or other target prediction software) are notoriously unreliable. Unless accompanied by experimental validation, these analyses are merely suggestive. Furthermore the details regarding how the IntaRNA analysis was performed are unclear. Consequently, I suggest removing section 3.6 from the results section. Some of the more interesting predicted interactions could be mentioned in the discussion section.

The predicted sRNA fragments were aligned using IntaRNA with the default settings. IntaRNA predicts interacting regions between two RNA molecules by incorporating the accessibility of both interaction sites and the presence of a seed interaction. We deleted the section 3.6 and moved some of the interesting, predicted interactions to the discussion section.

Line 401: Why were only 2 sRNAs investigated?

The reason why we selected the two specific sRNAs for investigation is that they could interact with known virulence-related genes in *H. somni*. These two were the most-promising candidates.

Line 410: What is the significance of the nucleotide residues in bold highlighted with an arrow in figure 6B and figure 6C?

The nucleotides in bold represent additional transcriptional start sites upstream of the transcriptional start site identified by RLM-RACE, which is indicated by the ‘arrow’. We have included it in the figure legend 4 (new figure no.).

Line 419: In the EMSA experiments shown in Fig 7, why are two bands of differing sizes detected for the *in vitro* transcribed RNA probes in all three experiments? Were the *in vitro* transcribed RNAs purified and DNAse treated? How was removal of DNA confirmed? Also it would be useful to include labels indicating the size and identity of each RNA molecule in Fig 7.

Yes, the *in vitro* transcribed probes were DNase treated, and the removal of DNA was confirmed by gel electrophoresis. Of the two bands of differing sizes detected in the *in vitro* transcribed RNA probes, the upper one is due to secondary structure formation. The reference figure indicating the secondary structure formation is given below. (Ref. Cheunim T et al. 2008, Virus Research 131(2-5):189-98).

**Secondary structure formation**

Fig. 4. hnRNP A1 binds the HPV16 LRE. (A) Electrophoretic mobility shift assay showing interaction of hnRNP A1 and LRE RNA in HeLa and W12 cells. Free probe and the RNA/protein complexes formed are indicated. Asterisks and an arrowhead indicate RNA/protein complexes that have been supershifted due to binding of the anti-hnRNP A1 antibody in tracks 3 and 5. Track 1, [α-32 P] rUTP-labelled LRE RNA probe alone (the LRE RNA is a stem loop structure (Cumming et al., 2003); the upper band is due to secondary structure formation).

We have indicated the size and identity of each RNA free probe in Fig 5 (new figure no.).

Line 431: “Once the 5’ and 3’ termini of the sRNA transcripts were assigned by RLM-RACE…” Identification of 3’ ends by RACE was not carried out. Please clarify this statement and/or add the 3’ RACE data.

We have not done 3’-RACE to identify the 3’ end of sRNAs. We have modified the sentence in the text.

Fig 8: The figure provided is extremely low resolution and blurry. Please provide high quality figures. Also the two individual RNAs should be labeled in the diagram.

We have modified Fig. 6 (new figure no.) and labelled the individual sRNAs.

Line 436: “The complex RNA-fold structures containing several stem loops in conjunction with the multiple target genes predicted by IntaRNA [40] is indicative of the potential of these sRNAs to control diverse biological processes.” This statement is a bit of an overreach and should be removed.

We have deleted the above-mentioned sentence in the revised manuscript.

Line 474: “The secondary structure (containing AU-rich regions and several stem loops) of the selected sRNAs confirms…” Please change the word “confirms” as the predicted secondary structures by themselves do not conclusively demonstrate anything.

We have changed “confirmed” to “indicated”.

Line 484 capitalize the G in gram negative. Same thing on line 494

We have modified it.

Line 485 “including pH”? Which pH are they referring to?

Technically, gram-negative does not refer to the inventor, Dr. Gram, as Gram strain does. Nonetheless, we have capitalized Gram everywhere as suggested.

Lines 480 to 505: This entire section of the discussion seems superfluous. It does not discuss any of the results generated in this manuscript, rather it discusses the roles of some small RNA's in other bacteria. It is unclear why this is included or if it is necessary.

We were trying to suggest the functional aspects of the sRNAs identified in this study by discussing that of their homologues well-studied in other bacteria. We have modified this section and made it more concise.

The authors should provide a more in-depth discussion of the similarities and differences between the sRNAs identified in this study and the study of Kumar et al (reference 8). For example, were the growth conditions similar? Can they speculate why certain sRNAs were identified in one study over another?

The differences between the sRNAs identified in our study with that of Kumar et al. could be due to the difference in culture conditions for the RNA extraction and the RNA type used for sequencing. In the study by Kumar et al., the *H. somni* 2336 cells were grown in Trypticase Soy Agar (TSA)-blood agar plates (with 5% sheep red blood cells) overnight, and the cells were washed with brain heart infusion (BHI) broth followed by total RNA isolation. Then, the mRNA enrichment was carried out using MICROBExpress. This enriched mRNA was sequenced, mapped, and intergenic regions were identified. The intergenic regions were analyzed to identify novel small RNAs. In our study, planktonic cultures of *H. somni* 2336 were cultured in Columbia broth (BD DifcoTM) with required growth supplements until stationary phase (reportedly higher production of Hfq). The cell extract was prepared and interacted with anti-Hfq IgG tagged magnetic beads to isolate Hfq and associated sRNAs. The purified sRNAs were directly used for sequencing followed by mapping and analysis to identify intergenic regions. Thus, in our study, we focused only on Hfq-associated sRNAs. However, the study by Kumar et al. reported the whole small RNA profile including the Hfq-associated sRNAs in *H. somni*. The data from Kumar et al., identified a number of sRNAs that were not detected in our study. In addition, there are sRNAs found in our study that were not detected by Kumar et al. The sRNAs isrK, tmRNA, gcvB, and those involved in the metabolism of lysine and glycine were identified in both studies, indicating their role in growth under both the culture conditions. We have modified the discussion section of the revised manuscript to indicate these differences.

While revising your submission, please upload your figure files to the Preflight Analysis and Conversion Engine (PACE) digital diagnostic tool, [https://pacev2.apexcovantage.com/](https://urldefense.com/v3/__https:/pacev2.apexcovantage.com/__;!!DeIc-uvKXH9G!68J5nNQLE7Jna-zZhAbUkpVyk1fGbQ64bS_dcR5W9ADLfDWaJYL1w68Zd2-Np7r69XEVSiVsOuOZPpqnDsll$). PACE helps ensure that figures meet PLOS requirements. To use PACE, you must first register as a user. Registration is free. Then, login and navigate to the UPLOAD tab, where you will find detailed instructions on how to use the tool. If you encounter any issues or have any questions when using PACE, please email PLOS at [figures@plos.org](mailto:figures@plos.org). Please note that Supporting Information files do not need this step.

We have used PACE to ensure that the figures in the revised manuscript meet PLOS requirements.
