# Supplementary material for: Identification and initial characterization of Hfq-associated sRNAs in Histophilus somni strain 2336
Source: PLoS One. 2023 May 23;18(5):e0286158. doi: 10.1371/journal.pone.0286158 (PMC10204968; doi:10.1371/journal.pone.0286158)
Supplement: Attachment — Submitted filename: Response to Reviewers second submision.docx [file pone.0286158.s004.docx]

**Response to Reviewer’s Comments**

Below are our responses (in red) to each of the reviewer’s comments/concerns. The manuscript has been revised accordingly based on these responses.

Reviewer #1: Thank you for your corrections. You think that you misunderstand my comment about the 'title'. This was specifically for Fig 1 (that has been removed). For me the original title of the manuscript was good.

We have changed the title back to the original title in this revised manuscript.

Reviewer #2: This is my second time reviewing this manuscript. While most of my previous comments have been sufficiently addressed some have not. There are also a number of outstanding issues with the manuscript that need to be addressed before it can be considered for publication.

Issues

Line 56: “provide improved understanding of the differences…”

We have corrected it.

Line 221: “the miRNA…” should this be mRNA or RNA?

We have corrected it as RNA.

Line 245. EMSA's were carried out for three sRNAs however in the materials and methods section no oligos are given for the in vitro transcription reaction using HS9

We have included the oligo information for HS9 in Table 1.

Line 265 says six independent sRNA preparations were used in deep sequencing reactions, but it is still not clear if these samples were individually barcoded and sequenced or if they were pooled and sequenced as one sample. The RNAseq data deposited online appears to only contain one data set so it would appear that all six samples were pooled and treated as one. This point was made in my original review and in response a table was included in the response to reviewer’s comments document showing the number of reads in each of the 6 samples. This table should be included in the manuscript. There is still a large discrepancy regarding the number of reads generated (see below) and the fact that the deposited data appears to be one dataset.

We have included the RNA sequence summary as Table 2 in the revised manuscript. The data deposited online are the results from sample GSF2751-sRNA_Hfq-8. The discrepancy regarding the number of reads is addressed below in the next comment. We have modified the text in the revised manuscript as well.

Line 271 says 20 million reads were generated however the RNAseq data deposited online contains only 2.4 million reads? The table provided in the response to reviewer’s comments also appear to contain less than 2.4 million reads?

20 million reads is the combined total number of reads generated from all 6 sRNA samples. Sorry for this reporting error. We have modified the text accordingly in the revised manuscript. The table below (portion of Table 2 now in the text) shows the number of reads generated from each sRNA sample and their total (20.99 M). The RNAseq data deposited online (from sample GSF2751-sRNA_Hfq-8) contains 2.4 M reads. All 6 samples are repetitions of sequences from the same strain, so GSF2751-sRNA Hfq-8 is a representative sample. The last column in Table 2 in the revised manuscript is the number of mapped reads.

| **Sample** | **Barcode sequence** | **PF Clusters** |  |  |
| --- | --- | --- | --- | --- |
|  |  |  |  |  |
| GSF2751-sRNA_Hfq-1 | CTATACAT | 4,416,420 |  |  |
| GSF2751-sRNA_Hfq-11 | TCGGCAAT | 2,667,639 |  |  |
| GSF2751-sRNA_Hfq-3 | CTCAGAAT | 5,000,014 |  |  |
| GSF2751-sRNA_Hfq-7 | GACGACAT | 3,411,220 |  |  |
| GSF2751-sRNA_Hfq-8 | TAATCGAT | 2,413,457 |  |  |
| GSF2751-sRNA_Hfq-9 | TCGAAGAT | 3,090,434 |  |  |
|  | Total: | 20,999,184 |  |  |

Fig 1. The figure legend for figure one is still incomplete. Line 300 says “the six black lines below it represents the six frame translation of the reference sequence” yet each black line is shown as one continuous line. What is the six-frame translation of the reference sequence the authors refer to? The description of other six frame translation (at the top of the figures, line 298-299) is also unclear. I made this point in my original review and it was not addressed sufficiently. What does the orange line represent? For HS97 which of the three regions that have transcript mapping to them represents the small RNA?

Below is the zoomed image of the six-frame translation (forward three frames and reverse three frames) of the *H. somni* genome reference sequence. The images in Fig. 1 was zoomed in to include more features including the whole sRNA regions which lead to the six frame translations showing as black lines.

The six-frame translation of the reference sequence refers to the translation of the *H. somni* 2336 genome in three overlapping reading frames in the forward direction and the complementary strand in the reverse direction.

Below is the zoomed image of the six-frame translation (forward three frames and reverse three frames) of the consensus sequence. The consensus sequence shows which residues are conserved (are always the same) and which residues are variable once we align the sequences to the reference genome sequence. The consensus is constructed from the most frequent residues at each site in the alignment.

If needed, we could remove the six-frame translation details of the reference genome sequence and the consensus sequence and include a revised version of Fig. 1.

The orange lines in Fig. 1 represent the maximum coverage for the RNA sequence. For HS97, the first region that has the transcript mapping represents the sRNA.

We have modified the Fig. 1 legend and the representative zoomed images of the six-frame translation of reference sequence and consensus sequence are shown as supporting information (S2 Supporting Information).

Line 341-342 (and elsewhere) the authors refer to checking the relative expression level of the candidate sRNA's based on the sequencing data however no RNAseq whole transcriptome sequencing data is presented in the manuscript. What data are they referring to? This data should be made available and deposited online.

The sRNA candidates were manually checked for their relative read coverage level in the sequencing data. The sRNAs with high read coverage were included for further analysis. We have modified the sentences in the revised manuscript.

Line 349 “Multi sequence analysis indicated that many of these sRNAs may interact with the Sigma 54 transcription factor”. Are the authors suggesting that these sRNAs interact with the messenger RNA encoding Sigma 54 or are they suggesting that the Sigma 54 protein directly binds to the SRNAs? Based on the data presented in table 3 it would appear that the latter is the case i.e they suggest that Sigma 54 protein binds to the RNAs. Are the authors suggesting that the Sigma 54 consensus binding site for DNA would be the same for single stranded RNA molecules? Is there any evidence for this?

Yes, we propose that the Sigma 54 protein binds to the sRNA regions in the genomic DNA, and not binding directly to the sRNAs. We have modified the Table 3 title and text in the revised manuscript.

Line 364 states that the selected eight sRNA candidates were chosen based on their predicted interaction with known biofilm and virulence related genes. However, on line 343 it states that the 8 sRNA candidates were selected based on preliminary bioinformatic analysis and the fact that they were highly expressed in the sequencing data. Can the authors resolve this inconsistency?

We have modified the text in the revised manuscript under “Northern blotting to verify selected sRNA candidates” to clarify that the selected sRNA candidates were chosen based on preliminary bioinformatic analysis and the relatively higher read coverage in the sequencing data.

Like 366 “based on their interaction with known virulence or biofilm genes” ... Are the authors referring to experimentally demonstrated interactions or bioinformatically predicted interactions. No reference is given?

Referring to bioinformatics predictions. We have modified the sentence in the revised manuscript.

Line 364 to 368 the rationale for going from 8 candidates to three candidates to two candidates is poorly described. No reason is given for the reduction from three to two candidate sRNAs.

Eight candidates were selected based on preliminary bioinformatics analysis and their relatively higher read coverage in the sequencing data. Out of the eight, three candidates were further selected based on their predicted interactions with known biofilm or virulence genes in *H. somni*. Our initial goal was to over-express and further characterize two of the most-promising sRNAs, and that’s why we selected only two sRNAs in the later part of our studies including determination of the transcriptional start site, prediction of secondary structures of selected sRNAs etc. We have modified the text in the revised manuscript and deleted the sentence “The two most promising candidates (HS79 and HS97) were selected for further analysis.”

Line 368 clearly states that the two most promising candidates (HS79 and HS97) were selected for further analysis…including northern blotting… yet then immediately on line 371 it's states that northern blocks were also performed for the third candidate HS9 (and included in Fig 3). Furthermore, in the materials and methods section (Table 1) no sequence is given for the probe used to detect HS9 by northern blot.

We have modified the text in the revised manuscript. The sentence stating that two of the most promising candidates (HS79 and HS97) were selected for further analysis under ‘Northern blotting to verify selected sRNA candidates’ has been deleted.. We have added the oligo details for HS9 for Northern blotting to Table 1.

In Figure 4 please indicate in panels B&C which one refers to HS79 and which refers to HS97

We have modified Fig. 4 to indicate the panels for HS79 and HS97.

Line 486 and line 489: gcvB/GcvB is formatted differently. Please be consistent.

We have corrected it in the revised manuscript.

Line 496 “Target predictions for the select sRNAs”... Which select RNAs?

Target predictions were carried out for a number of sRNAs including the 8 sRNAs with relatively high read coverage in the sequencing data. We have modified the sentence in the revised manuscript.

Line 503-504 should read “in addition some sRNAs were predicted to interact with their target genes...”

We have modified the sentence in the revised manuscript.

Line 514 should read “further studies on the sRNAs that are predicted to be associated with these…”

We have modified the sentence in the revised manuscript.

Line 530 “many of the sRNA candidates could potentially interact with genes”

We have modified the sentence in the revised manuscript.
